# A Recombinant *Anticarsia gemmatalis* MNPV Harboring *chiA* and *v-cath* Genes from *Choristoneura fumiferana* Defective NPV Induce Host Liquefaction and Increased Insecticidal Activity

**DOI:** 10.1371/journal.pone.0074592

**Published:** 2013-09-25

**Authors:** Anabele Azevedo Lima, Clara Wandenkolck Silva Aragão, Maria Elita Batista de Castro, Juliana Velasco de Castro Oliveira, Daniel Ricardo Sosa Gómez, Bergmann Morais Ribeiro

**Affiliations:** 1 Graduate Program in Molecular Pathology, University of Brasília, Brasília, Distrito Federal, Brazil; 2 Embrapa Genetic Resources and Biotechnology, Brasília, Distrito Federal, Brazil; 3 Department of Microbiology, University of São Paulo, São Paulo, Brazil; 4 Embrapa Soybean, Londrina, Paraná, Brazil; Universidad Pública de Navarra, Spain

## Abstract

One of the interesting features of *Anticarsia gemmatalis multiple nucleopolyhedrovirus* isolate 2D (AgMNPV-2D) genome is the absence of chitinase (*chiA*) and cathepsin (*v*-*cath*) genes. This characteristic may be responsible for the lack of liquefaction and melanization in *A. gemmatalis* larvae killed by AgMNPV-2D infection. This study aimed to test the hypothesis that CHIA and V-CATH proteins from *Choristonera fumiferana* DEF *multiple nucleopolyhedrovirus* (CfDEFNPV) are able to liquefy and melanize the cuticle of *A. gemmatalis* larvae infected by a recombinant AgMNPV containing *chiA* and *v*-*cath* genes inserted in its genome. A fragment from the CfDefNPV genome containing *chiA* and *v*-*cath* genes was inserted into the genome of AgMNPV-2D. The recombinant virus (vAgp2100Cf.chiA/v-cath) was purified and used to infect insect cells and larvae. Transcripts of *v*-*cath* and *chiA* genes were detected along the infection of insect cells by qRT-PCR, from early to late phases of infection. The analysis of *A. gemmatalis* larvae killed by vAgp2100Cf.chiA/v-cath infection confirmed the hypothesis proposed. The vAgp2100Cf.chiA/v-cath showed higher insecticidal activity against third instar *A. gemmatalis* larvae when compared to AgMNPV-2D. The mean time to death was also lower for the vAgp2100Cf.chiA/v-cath when compared to AgMNPV-2D at 10 days post infection. Occlusion body production was higher in *A. gemmatalis* larvae infected with vAgp2100Cf.chiA/v-cath when compared to AgMNPV-2D. Enzyme assays showed higher chitinase and cysteine protease activities in insect cells and insects infected with vAgp2100Cf.chiA/v-cath when compared to AgMNPV-2D. The introduction of *chiA* and *v-cath* genes into the genome of AgMNPV improves its insecticidal activity against *A. gemmatalis* larvae and this recombinant virus could be used as an alternative to the wild type virus to control this important insect pest.

## Introduction

Brazil is considered the second largest soybean producer and the third largest exporter of agricultural products in the world [1]. Insect pest control has mainly been done by chemical insecticides. An alternative to the use of chemical pesticides in the crops to control insect pests is the use of biological agents, such as the baculoviruses [2]. These viruses contain rod-shaped virions and a large, circular, super coiled double-stranded DNA genome ranging from 80 to 200 kilobases (kb) [3]. The *Baculoviridae* family is divided into four genera: *Alphabaculovirus* (lepidopteran-specific nucleopolyhedrovirus, NPV), *Betabaculovirus* (lepidopteran-specific granulovirus, GV), *Gammabaculovirus* (hymenopteran-specific nucleopolyhedrovirus, NPV), and *Deltabaculovirus* (dipteran-specific nucleopolyhedrovirus, NPV) [4], [5].

A peculiarity of baculoviruses is the production of two phenotypically distinct viruses in a single cycle of infection: the budded viruses (BVs), which are responsible for the systemic infection within the host, from cell to cell; and the occlusion-derived viruses (ODVs), which are occluded in a proteinaceous occlusion body (OB), also known as polyhedra, responsible for horizontal transmission from insect to insect [6]. In the final stages of infection, infected insects become weakened by losing its motor and feeding capacity, the cuticle becomes whitened due to accumulation of large amounts of OBs in the cell nucleus of epidermal and fat body cells [7]. When the insect larvae infected by the virus dies, its tegument darkens (melanizes) and disintegrates easily, releasing large amounts of OBs in the environment, serving as an inoculum source for the infection of other insect hosts [8]. The disintegration of the host is facilitated by the synergistic interaction between the viral proteins V-CATH (a cysteine protease) and CHIA (a viral chitinase) which are codified by two genes present in several baculovirus genomes [9], [10].

The baculovirus *Anticarsia gemmatalis multiple nucleopolyhedrovirus* (AgMNPV) is a bioinsecticide used in large scale in Brazil to control populations of velvetbean caterpillar, *Anticarsia gemmatalis* (Hübner, [1818]) (Lepidoptera: Noctuidae), a major defoliator of soybean fields. After the completion of the AgMNPV genome, its initial analysis showed the absence of the auxiliary genes *chiA* and *v*-*cath* that are usually found in the genomes of most *Alphabaculoviruses* sequenced to date [11]. Baculoviruses genomes encode auxiliary genes that are not essential for viral replication, but they confer, however, selective advantage to the virus. The expression of these two proteins occurs in the late phase of virus infection, and synergism between these two proteins promotes the liquefaction of the host body [12]. The absence of *chiA* and *v-cath* genes in the AgMNPV genome may be the cause for the lack of body liquefaction after death of AgMNPV-infected larvae [11]. This feature is important for the biological control program because it facilitates larvae collection after death and the consequent formulation of this bioinsecticide.

CHIA protein from AcMNPV presents exo and endo chitinase activity [12], and it is located in the endoplasmic reticulum (ER) of infected insect cells probably due to the presence of a C-terminal retention motif (KDEL). The chitinase activity of AcMNPV CHIA is essential for host liquefaction [13], [14]. AcMNPV CHIA was also shown to be involved in the processing of V-CATH encoded by the virus [15]. V-CATH is synthesized as a proenzyme, and it is proteolytically cleaved to its mature form in the later stages of the infection [15]. The proteolytic cleavage of pro-cathepsin seems to be assisted by CHIA, which may act as a chaperone in the ER [16]. V-CATH is a papain type cysteine protease which is homologous to a lysosomal cysteine protease, cathepsin L [9], [10]. In AcMNPV, this protein presents precursor forms of approximately 35.5 and 32.0 kDa (pro-V-CATH) which are both processed to a mature form of 27.5 kDa similarly to cathepsin L [10]. However, Hom and Volkman 2000 [16] found that in the absence of *chiA* gene in the genome of AcMNPV, proV-CATH is not processed in its mature form, forming insoluble aggregates within infected cells. Studies have shown that BmNPV and AcMNPV mutants that are unable to produce V-CATH do not cause terminal liquefaction of the infected host insect [9], [10], [17].

Considering that most Alphabaculoviruses have in their genomes the *chiA* and *v-cath* genes, and given the importance of the action of these proteins in the virus-host interaction resulting the insect´s final liquefaction and further spread of viral progeny, this study proposed to test the hypothesis that the expression of proteins CHIA and V-CATH from CfDefNPV in *A. gemmatalis* larvae during infection by a recombinant AgMNPV induces liquefaction of the insect tegument and its melanization.

## Materials and Methods

### Insect, cell lines and viruses

*Anticarsia gemmatalis* (Hübner, [1818]) larvae were obtained from Embrapa Genetic Resources and Biotechnology insect rearing laboratory (Brasilia, Brazil) and used for production and purification of occlusion bodies (OBs) of wild-type and recombinant viruses, and bioassays. For infection and viral titer experiments, insect cell lines Sf9 from *Spodoptera frugiperda* (SF-9 *ATCC® CRL-1711*), and UFL-AG-286 derived from *A. gemmatalis* [18] were used. Virus titration was performed by the TCID_50_ method [19]. Cells were maintained in TC-100 medium (GIBCO-BRL) supplemented with 10% fetal bovine serum at 28°C. The viruses used in this study were AgMNPV-2D [20], AgMNPV-LDB80 (an AgMNPV isolate from Embrapa Soybean, Londrina, Brazil) and AcMNPV L-1 [21]. The recombinant vAgGalA2, is an AgMNPV-2D-derived virus and has the *E. coli lac-Z* reporter gene in the locus of the polyhedrin gene (*polh*) under the *polh* promoter [22].

### Construction of plasmids containing *chi*A and *v-cath* genes

Plasmid pCfDefNPVHindIIIQ, kindly supplied by Dr. Basil Arif (Great Lakes Forestry Centre, Canadian Forest Service, Sault Sainte Marie, Ontario, Canada), was digested with the restriction enzyme *Hin*dIII (Promega) [23]. A 3.236 bp fragment containing the complete ORF of the *chi*A e *v-cath* genes and part of its upstream and downstream flanking regions was extracted from the agarose gel and later purified according to the PureLinkTM Quick Gel Extraction Kit protocol (Invitrogen). The DNA cassette was inserted into the transfer vector p2100*Hin*dIII previously digested with *Hin*dIII, dephosphorylated with CIAP enzyme – Calf Intestinal Alkaline Phosphatase (Promega), and treated with T4 DNA ligase (Promega) following the manufacturer's instructions. The ligation was transformed into competent *E. coli* DH5α (Invitrogen), according to the heat shock method [23]. The isolated recombinant *E. coli* was amplified in L-Broth medium, followed by plasmid DNA extraction on a large scale according to the alkaline lysis protocol [23]. The p2100 vector was constructed as described in Cordeiro et al. (2008) [24]. In brief, this vector was constructed from PCR amplifications using the AgMNPV HindIII-G DNA fragment cloned into plasmid pGEM3Z (Maruniak et al. 1999), generating two fragments of 682 and 1439 bp, using the oligonucleotides pairs polAgR/SphIF and polAgF/PstIR, respectively (Table S1). A new PCR was performed with fragments of 682 and 1439 bp, using the oligonucleotide pair polAgF/polAgR generating a 2100 bp fragment which was then cloned into the pGEM-T vector, resulting in the plasmid p2100. A *Hin*dIII linker (BioLabs) was inserted into the *Eco*RV restriction site of plasmid p2100 at 100 bp upstream of the *polh* ATG in order to facilitate the cloning of the chiA/v-cath gene cassette.

To confirm the introduction of *chiA* and *v-cath* genes into the recombinant plasmid, a plasmid DNA digestion with the restriction enzyme *Hin*dIII (Promega) was carried out as described above. Besides the digestion, a PCR was performed to confirm the insertion of the genes in the recombinant plasmid, using the recombinant plasmid DNA as template and specific primers for *chiA*, QUITCfF and QUITCfR and *v-cath*, CATHCf F CATHCf R genes (Table S1) from CfDefNPV, which were designed from the gene sequences deposited in the Genbank database (access number AY327402). Amplifications were carried out by using *Taq* DNA polymerase (Invitrogen) following the manufacturer's instructions (Invitrogen) in a Perkin Elmer thermocycler (GeneAmp PCR System 2400) set for 30 cycles, each consisting on the following sequence: initial denaturation at 95°C for 5 min; amplification cycle: denaturation at 95°C for 1 min, annealing at 56°C for 1 min and 30 s, extension at 72°C for 1 min and 30 sec; and final extension at 72°C for 7 min.

### Generation of recombinant AgMNPV containing the *chi*A and *v-cath* genes

The recombinant p2100Cf.chiA/v-cath plasmid DNA (1 μg) was co-transfected with the DNA from the recombinant virus vAgGalA2 (0.5 μg) in UFL-AG-286 cells (1×10^6^ cells) using liposomes (Cellfectin®), following the manufacturer's instructions (Invitrogen). Seven days after co-transfection, the transfected cells supernatant was used to isolate the recombinant baculovirus containing the *chiA/v-cath* genes by serial dilution in 96 well plates [25]. Since the p2100Cf.chiA/v-cath plasmid contains besides the *chiA* and *v-cath* genes, the *polh* gene from AgMNPV, upon homologous recombination, the *polh* gene is introduced into the genome of the vAgGalA2 virus and the recombinant virus can be easily seen by the presence of occlusion bodies under light microscopy. Seven isolation cycles in 96 well plates were required for the proper isolation of the recombinant virus. The recombinant virus was used to infect UFL-AG-286 cells (1×10^7^ cells), and after seven days p.i., BVs in the supernatant were purified, and viral DNA was subsequently extracted [25].

To confirm the presence of *chiA*/*v*-*cath* genes in the recombinant viral genome, a PCR reaction was performed using the purified viral DNA and specific oligonucleotides (QUITCfF/QUITCfR CATHCfF and/CATHCfR) as described above (Table S1). Besides the PCR reaction, the DNA from the wild virus AgMNPV-2D and from the recombinant viruses were digested with *Hin*dIII [23]. After the electrophoresis, the gel was transferred to a nylon membrane (0.2 μm, Sigma) by Southern blot using a Vacu-Aid apparatus (Hybaid) following the manufactureŕs instructions. A nonradioactive probe was constructed by PCR using DNA from the recombinant plasmid containing the *chiA*/*v-cath* genes and oligonucleotides QUITCfF and QUITCfR, following the instructions from the DIG DNA Labelling and Detection Kit (Boehring Mannheim). The membrane was then hybridized with the non-radioactive probe, and the detection of the hybridized fragments on the membrane was performed with NBT/BCIP substrate following the manufacturer's instructions (Zymed – Invitrogen).

### Real time PCR

To analyze the temporal expression of *v-cath* and *chi*A genes during insect cells infection with AcMNPV and vAgp2100Cf.chiA/*v-cath*, specific oligonucleotides were designed to amplify fragments corresponding to the related genes in a PCR (Table S1). Each reaction was performed in a final volume of 50 μL, by containing 4 µL (5 ng/µL) of viral DNA, 20 pmol of each oligonucleotide, 0.4 mM of dNTPs, 1X reaction buffer, 2.0 mM MgCl_2_, and 1.5 U of Taq DNA polymerase (Invitrogen). The initial cycle used was 95°C for 2 min and 35 cycles of 95°C for 30 s, 52°C for 30 s and 72°C for 50 s, with a final extension at 72°C for 10 min.

To analyze the transcripts, Sf-9 and UFL-AG-286 cells (5×10^5^) were infected with each virus (10 pfu/cell). During different times after infection (0, 6, 12, 24, 48 and 72 h p.i.), cells were collected and RNA was extracted with RNeasy Plus kit (Qiagen) and subsequently treated with DNAse (using the DNA-free kit -Ambion) to remove any contaminating DNA present in the RNA solution. Then, the cDNA was synthesized with the SuperScriptIII RT kit (Invitrogen) and oligo (dT), following the manufacturer's instructions (Invitrogen). The synthesis was performed with 100 ng of DNase-treated RNA (NanoDrop ND-1000– NanoDrop Technologies) in a final volume of 120 µL. The cDNA was later subjected to real-time PCR for each gene [26]. The real-time PCR analysis was performed using fluorophore IQ™SYBR® Green Supermix (Bio-Rad) in the equipment Rotor-Gene™ 3000 (Corbett Research) with Rotor-Gene software version 6.1 (Corbett Research). The reactions were performed as follows: 1.0 µL of cDNA, 0.5 volumes of 2X IQ™ SYBR® Green Supermix (Bio-Rad), and 5 pmol of each specific oligonucleotide (AcQuitF/AcQuitR; AcCatF/AcCatR; CfQuitF/CfQuitR; CfCatF/CfCatR) to a final volume of 15 µL. The PCR program used was: 96°C for 2 min, followed by 40 cycles of 96°C for 30 s, 52°C for 30 s and 72°C for 40 s. A standard curve was obtained for each real time PCR product from each gene. The PCR products of known concentration were diluted serially in logarithmic order (base 10) and used for real time PCR analysis. The standard curve is based on the initial concentration of each dilution and on the number of required cycles to detect amplification. With this curve, it was possible to calculate the initial concentration of each gene according to each kinetics time. The data generated was compared and organized into a graph with number of copies/ng of total RNA in a logarithmic scale.

### Structural analysis of *A. gemmatalis* larvae infected with the recombinant virus

Third-instar *A. gemmatalis* larvae were infected in order to observe if the recombinant virus was able to cause liquefaction and melanization of the larvae at the end of infection. The infection was carried out by microinjection, on the second pair of legs, of about 10–50 μL of BV stock from AgMNPV and recombinant virus (10^8^ pfu/mL). Larvae were maintained at 25°C in plastic cups and fed on artificial diet [27]. The larvae were viewed and photographed daily until death.

### Purification of virus occlusion bodies

Third-instar *A. gemmatalis* larvae were infected via microinjection with AgMNPV and the recombinant virus was kept in plastic cups and fed on an artificial diet [27] as described above. After 90 h p.i., larvae were collected and macerated with 8 mL of homogenization buffer (1% ascorbic acid, 2% SDS, 0.01 M Tris pH 8.0, 0.001 M EDTA) and the purification of the occlusion bodies of each virus was performed according to O'Reilly et al., 1992 [25]). The direct counting of polyhedra was done in a light microscope (200×) using a hemacytometer (INLAB) [28]. Counting was performed in triplicate.

### Bioassays

Larvae of *A. gemmatalis* were analyzed by bioassays with OBs incorporated in the artificial diet [29]. Six dilutions were prepared at different concentrations: 4860, 1600, 540, 180, 60, and 20 OB/mL from the occlusion bodies of vAgp2100Cf.chiA/v-cath, AgMNPV-2D and AgMNPV-LDB80. The artificial diet was prepared [27] without formaldehyde, and cooled to 50°C. Still in the liquid stage, 10 mL of the virus suspension was mixed with 90 mL of artificial diet. Then, each mixture was poured into 10 mL plastic cups of about 50 mL (about 10 mL/cup). As a control, artificial diet was mixed only with sterile distilled water. Three third-instar larvae were placed per cup, using a total of 20 cups (60 larvae/concentration). The bioassays were carried out in a B.O.D. incubator at 26°C and photoperiod of 14 hours, and the mortality was recorded daily. The dose-mortality data were analyzed using Polo-Plus [30] and relative potencies calculated according to Robertson and Preisler (1992) [31]. Virus-infected *A. gemmatalis* larvae used in the bioassays were collected upon death and the amount of OBs per gram of dead larvae was counted using a Neubauer chamber with phase contrast microscope. Counting was performed in triplicate. Comparisons of the numbers of occlusion bodies ×10^9^ per g of larvae were done with parametric analysis ANOVA and means compared by Tukey test using SigmaPlot version 11.0, from Systat Software, Inc., San Jose California USA, www.sigmaplot.com.

### Chitinase activity assay

We tested the activity of chitinase present in purified polyhedra of AgMNPV and vAgp2100Cf.chiA/v-cath as well as in virus-infected UFL-AG-286 (1×10^7^) cells extracts using regenerated chitin [32] and 4-methylumbelliferyl-β-DN, N'-diacetylchitobioside [4MU-(GlcNAc) 2] (Sigma). The protein concentration in all samples was determined by the Bradford method [33]. Enzyme assays were performed in triplicates using 100 mg of protein.

The chitinase activity was determined by the DNS method [34], using chitin as substrate. About 100 mg of purified polyhedra protein from AgMNPV and vAgp2100Cf.chiA/v-cath were added to a solution containing 500 μL of regenerated chitin 0.5%, 250 μL of sodium acetate buffer 50 mM pH 5.2 and 1 mL sodium acetate buffer. The samples were incubated at 37°C for 16 h at 200 rpm (Thermo Fisher Science 491). After this incubation period, the samples were centrifuged at 10.000×g for 5 min and 250 μL of supernatant was collected and mixed with 750 μL DNS [34]. Then, the samples were boiled for 10 min and the amount of reducing sugar formed was estimated spectrophotometrically (SpectraMax, Molecular Devices Corporation, USA) at 550 nm. Regenerated chitin and distilled water (no enzyme) were used as controls.

The chitinase activity was also measured using a modified version of the method described by Trudel and Asselin [35]. UFL-AG-286 cells mock-infected and infected (10 pfu/cell) with the AgMNPV or vAgp2100Cf.chiA/v-cath viruses were collected at 72 h p.i. by centrifugation at 3.000×g for 10 min. The supernatant was removed and the cell pellet was washed twice with 1× PBS buffer (NaCl 137 mM, Na_2_HPO_4_ 10 mM, KCl 2.7 mM, and pH 7.4) then resuspended in 500 μL of 1× PBS buffer and stored at −80°C. Each sample (100 mg) was mixed in 350 μL Sodium phosphate buffer 10 mM pH 6.0 and 100 mL of substrate (5 mM 4 MU-(GlcNAc)_2_ in sodium phosphate buffer 10 mM pH 5.0). After incubation for 1 h at 37°C, the reaction was stopped by adding 1.0 mL of 0.25 M Na_2_CO_3_ and analysed in a spectrophotometer at 550 nm (SpectraMax, Molecular Devices Corporation, USA). Substrate in water (without enzyme) was used as control. Student's t-test was used to compare values obtained in the enzyme assay experiments.

### Chitinase gel activity assay

UFL-AG-286 cells (1×10^6^) were infected with AgMNPV and vAgp2100Cf.chiA/v-cath viruses (10 pfu/cell) and collected at 48 and 72 h p.i., washed 3 times with 1x PBS buffer (pH 7.4) and stored at −80°C. The samples (25 μg of each sample) were then subject to electrophoresis in a resolving 7.5% polyacrylamide gel under native conditions (without boiling) [36] and containing chitin glycol (1%). After electrophoresis, the gel was washed once with Triton X-100 (1%) and incubated for 24 h at 37°C with sodium acetate buffer pH 5.0 plus 0.1 M Triton X-100 (1%). After incubation, the gel was submerged for 10 min in freshly prepared developing solution of calcofluor white M2R (Sigma) (0.01%) in Tris-HCl 0.5 M pH 8.0 [35]. The developing solution was removed and the gel incubated for 1 h in distilled water at room temperature. Finally, the gel was exposed to UV light and photographed.

### Cysteine protease assay

Two types of substrates were used to measure the activity of cysteine protease. For assays with hemolymph from mock-infected and virus-infected insects and purified polyhedra (as described above) keratin blue was used as substrate (Keratin Azure, Sigma). At 96 h p.i., the hemolymph from 25 *A. gemmatalis* larvae (infected and uninfected as described above) was collected using a small hole made in the second pair of legs. The collected hemolymph was homogenized in anticoagulant buffer (98 mM NaOH, 186 mM NaCl, 1.7 mM EDTA, 41 mM citric acid, pH 4.5) for preventing melanization and then stored at −80°C until use. The assay was conducted in triplicate using 100 mg of protein present in each sample (hemolymph or polyhedra). Samples were incubated with 500 μL of reaction buffer (50 mM sodium phosphate pH 7.0, 0.1 M MgCl_2_ and 50 μg of keratin blue) for 3 h at 37°C. After incubation, the reaction was quenched with 100 mL of 10% TCA (Trichloroacetic acid, Sigma), kept on ice for 1 h, centrifuged at 10.000×g for 5 min. Five hundred μL of supernatant collected and transferred to a new microtube that was analyzed in a spectrophotometer (SpectraMax, Molecular Devices Corporation, USA) at an absorbance of 595 nm. Keratin blue in distilled water were used as control.

For assays with virus-infected and mock-infected insect cells extracts, Azocasein (Sigma) was used to detect proteolytic activity. UFL-AG-286 cells (1×10^8^) were infected with the wild type or recombinant virus (10 pfu/cell) and after 40 h p.i., total cell extracts were collected and centrifuged at 3.000×g for 10 min. The supernatant was removed and the cell pellet was washed twice with 1× PBS buffer (pH 7.4). The cells were homogenized in sodium phosphate buffer pH 6.8 and centrifuged again at 10.000×g for 20 min. After centrifugation, 250 μl of the supernatant was incubated with 400 µL of Azocasein (6 mg/ml in 100 mM succinic acid-NaOH, pH 4.1). The samples were incubated for 5 h at 37°C at 200 rpm. The reaction was stopped by adding 100 μL of trichloroacetic acid (TCA) 100%. The tubes were centrifuged at 12.000×g for 5 min and the absorbance of the supernatant measured in a spectrophotometer at 280 nm (SpectraMax, Molecular Devices Corporation, USA). Samples were analyzed in the presence and absence of protease inhibitor E-64 (2.8×10^−4^ M) (Sigma). Sample incubated without substrate was used as control. Student's t-test was used to compare values obtained in the enzyme assay experiments.

## Results

### Construction of AgMNPV recombinant virus containing the *chiA and v-cath* genes

**Figure 1:**
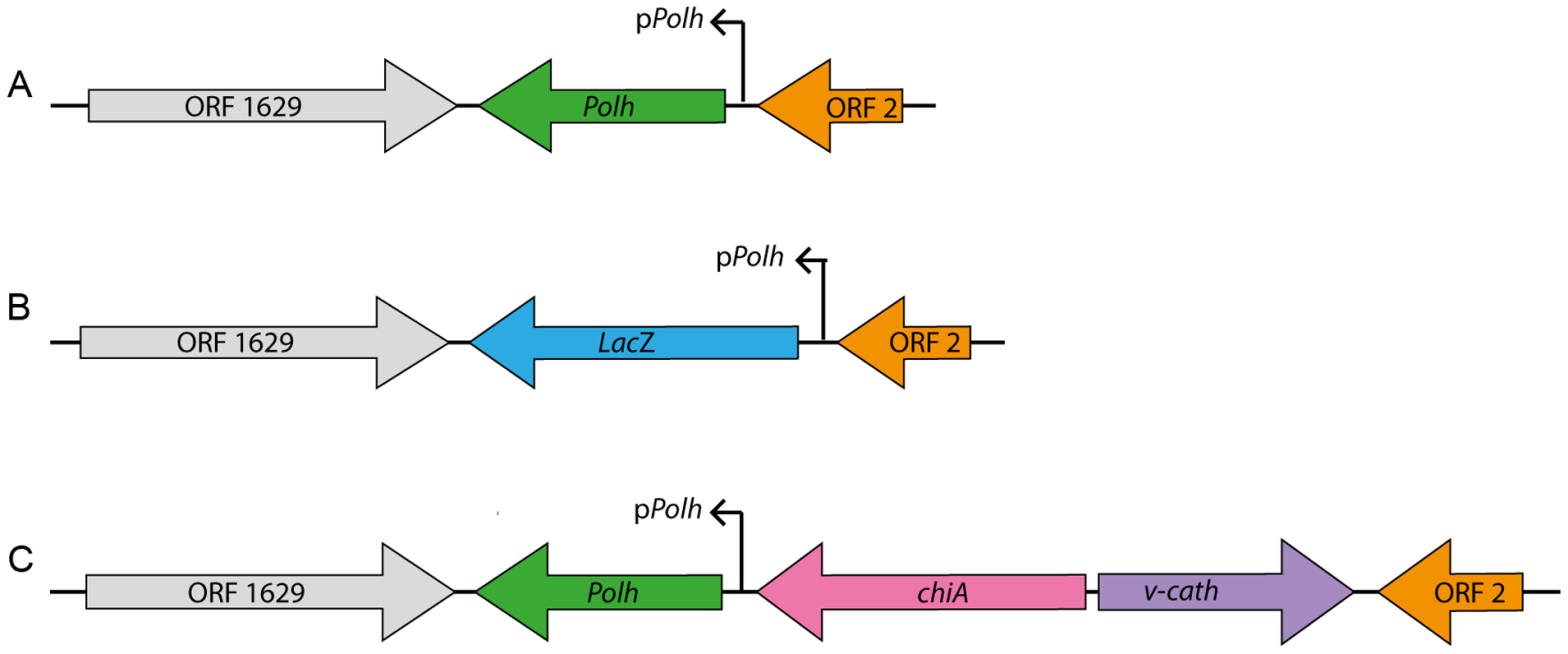
Schematic representation of the *polh* locus in AgMNPV and two recombinant viruses. The figure shows the orientation of *v-cath* and *chi*A genes, as well as the promoter and the *polh* gene and flanking ORFs (ORF 1629 and ORF2).

p2100Cf.chiA/v-cath plasmid DNA was cotransfected with DNA from the recombinant baculovirus vAgGalA2 in insect cells (UFL-AG-286). Inside the insect cells, the homologous recombination took place with the exchange of homologous regions between both DNAs from the vector plasmid and the viral genome, resulting in the recombinant virus vAgp2100Cf.chiA/v-cath. After virus isolation by serial dilution in 96 well plates [25], the confirmation of the gene insertion in the genome of AgMNPV (Figure 1) was performed by PCR using specific primers for the *chi*A e *v-cath* genes (data not shown), and also by Southern blot (Figure S1) using a nonradioactive DNA probe. After hybridization with the probe, the expected band size corresponding to the recombinant genes inserted into the baculovirus genome was detected (see supporting material).

### Transcription analysis of insect cells infected with AcMNPV and recombinant AgMNPV viruses

**Figure 2:**
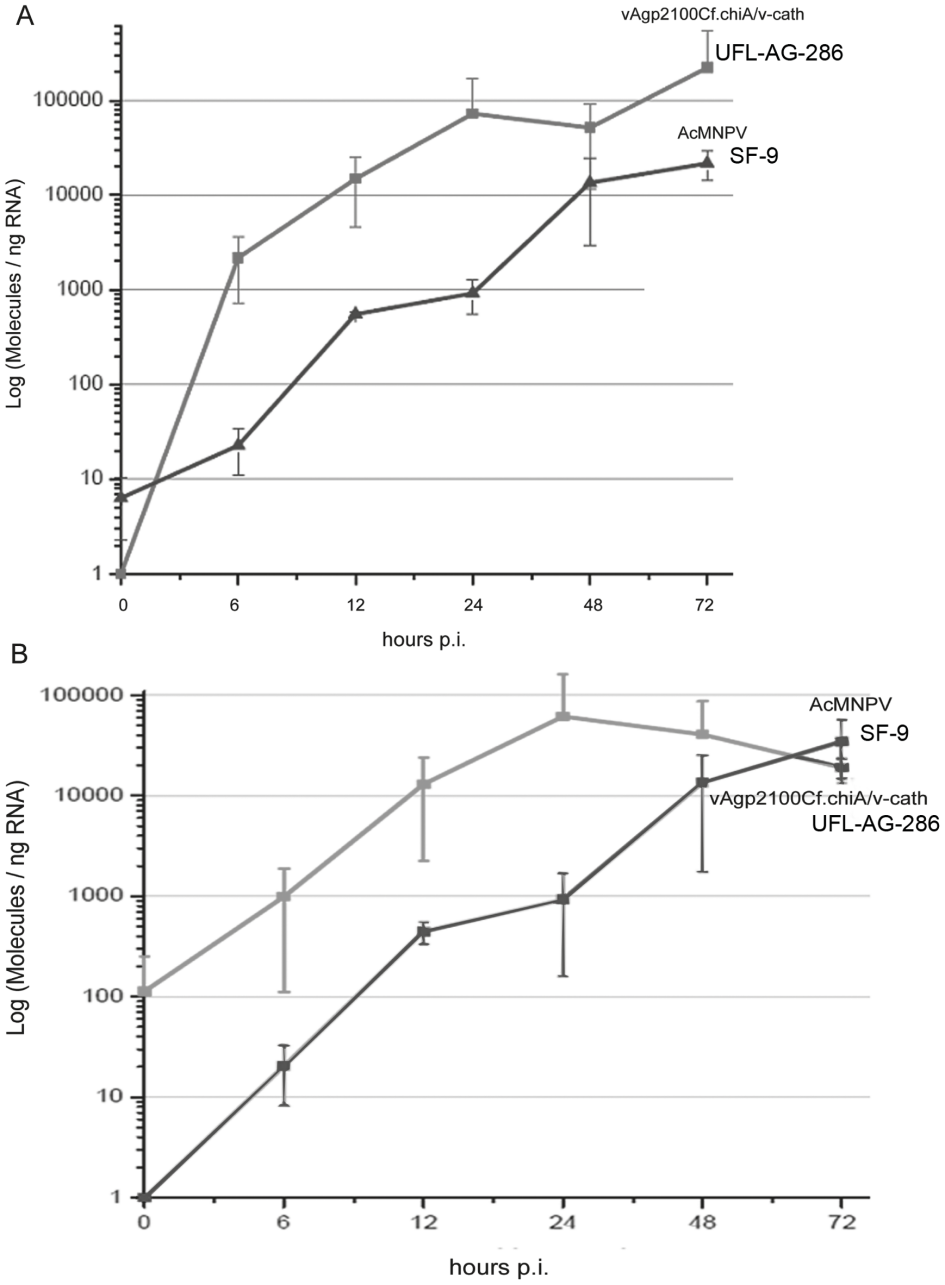
Average temporal expression of *v-cath and chiA genes* of AcMNPV and vAgp2100Cf.chiA/*v-cath*. qRT-PCR was used to analyze the temporal expression of *v-cath* gene during insect cells infection, Sf-9 and UFL-AG-286 cells, with AcMNPV and vAgp2100Cf.chiA/*v-cath*, respectively. The graphs show the amount of mRNA molecules per ng of total RNA of the *v-cath* (panel A – dark gray line) and *chiA* (panel B – light gray line) genes during infection of Sf-9 cells with the wild type virus (black line) and UFL-Ag-286 cells during infection with the recombinant virus (gray line) at different times post infection. Bars represent standard error.

UFL-AG-286 and SF-9 cells were infected with vAgp2100Cf.chiA/v-cath and AcMNPV, respectively, and collected at different times post infection (0, 6, 12, 24, 48, and 72 h p.i.). The mRNAs were extracted and used to construct cDNAs. These cDNAs were amplified by real time PCR with *chiA* and *v-cath*-specific oligonucleotides. The data generated were transformed into gene copies/ng of RNA, generating a graph of the temporal expression of *chi*A and *v-cath* genes (Figure 2A and B). The transcription profiles of *chi*A and *v-cath* genes present in the AcMNPV and in vAgp2100Cf.chiA/v-cath genomes were similar, during infection of insect cells (UFL-AG-286 for vAgp2100Cf.chiA/v-cath and Sf-9 for AcMNPV), with a progressive increase from 6 h p.i. until 48 h p.i. However, the transcription level of *chi*A decreased after 48 h p.i. in Sf-9 cells infected with AcMNPV, when compared to *chi*A expression in UFL-AG-286 infected with vAgp2100Cf.chiA/v-cath. The transcription of both genes in UFL-AG-286 cells indicates that the CfDefNPV gene promoters are functional in these cells. The average transcription in most of the different times analyzed was higher in the vAgp2100Cf.chiA/v-cath/UFL-AG-286 system than in the AcMNPV/Sf-9 system.

### Liquefaction of *A. gemmatalis* larvae infected with vAgp2100Cf.chiA/v-cath

**Figure 3:**
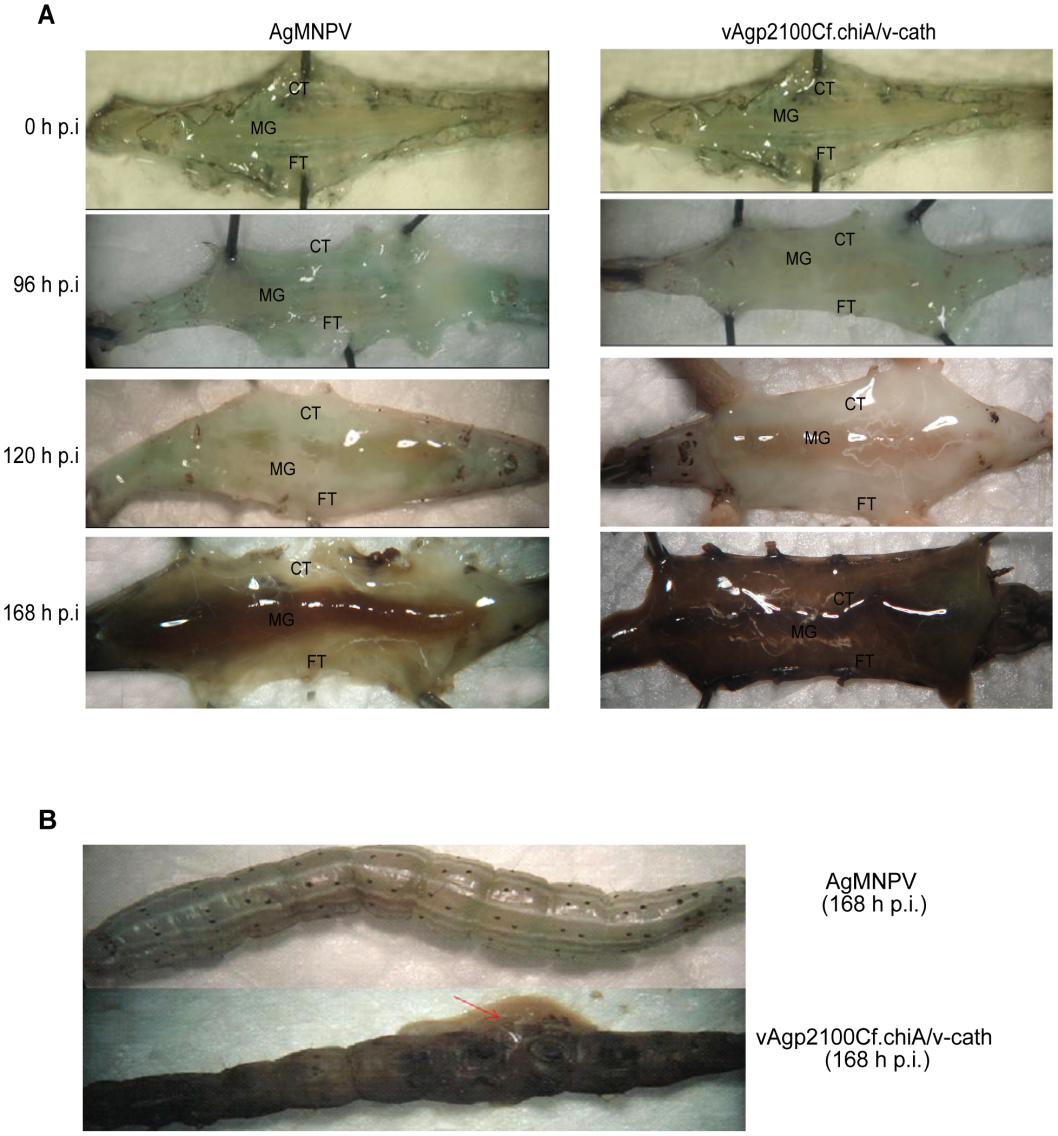
Effect caused by the wild type (AgMNPV) and recombinant (vAgp2100Cf.chiA/*v-cath*) viruses in *A. gemmatalis* larvae. (A) Structural analysis of internal tissues of larvae infected with AgMNPV and vAgp2100Cf.chiA/v-cath at different times post infection. MG – midgut, CT – cuticle; FT – fat tissue. (B) L. Larvae infected with wild type and recombinant virus at 168 h p.i., showing body liquefaction and melanized cuticle only in recombinant virus-infected larvae (vAgp2100Cf.chiA/v-cath).

*A. gemmatalis* larvae inoculated with AgMNPV and vAgp2100Cf.chiA/v-cath were dissected and their internal organs were observed at different times post infection (Figure 3A). AgMNPV-infected larvae showed internal tissues apparently intact, and there was no sign of melanization and degradation of the larval body cuticle. On the other hand, vAgp2100Cf.chiA/v-cath-infected larvae showed their internal organs completely liquefied, they also showed a remarkable cuticle degradation and melanization at 168 h p.i. (Figure 3B).

### Recombinant AgMNPV virus has increased insecticidal activity towards *A. gemmatalis* larvae

**Figure 4:**
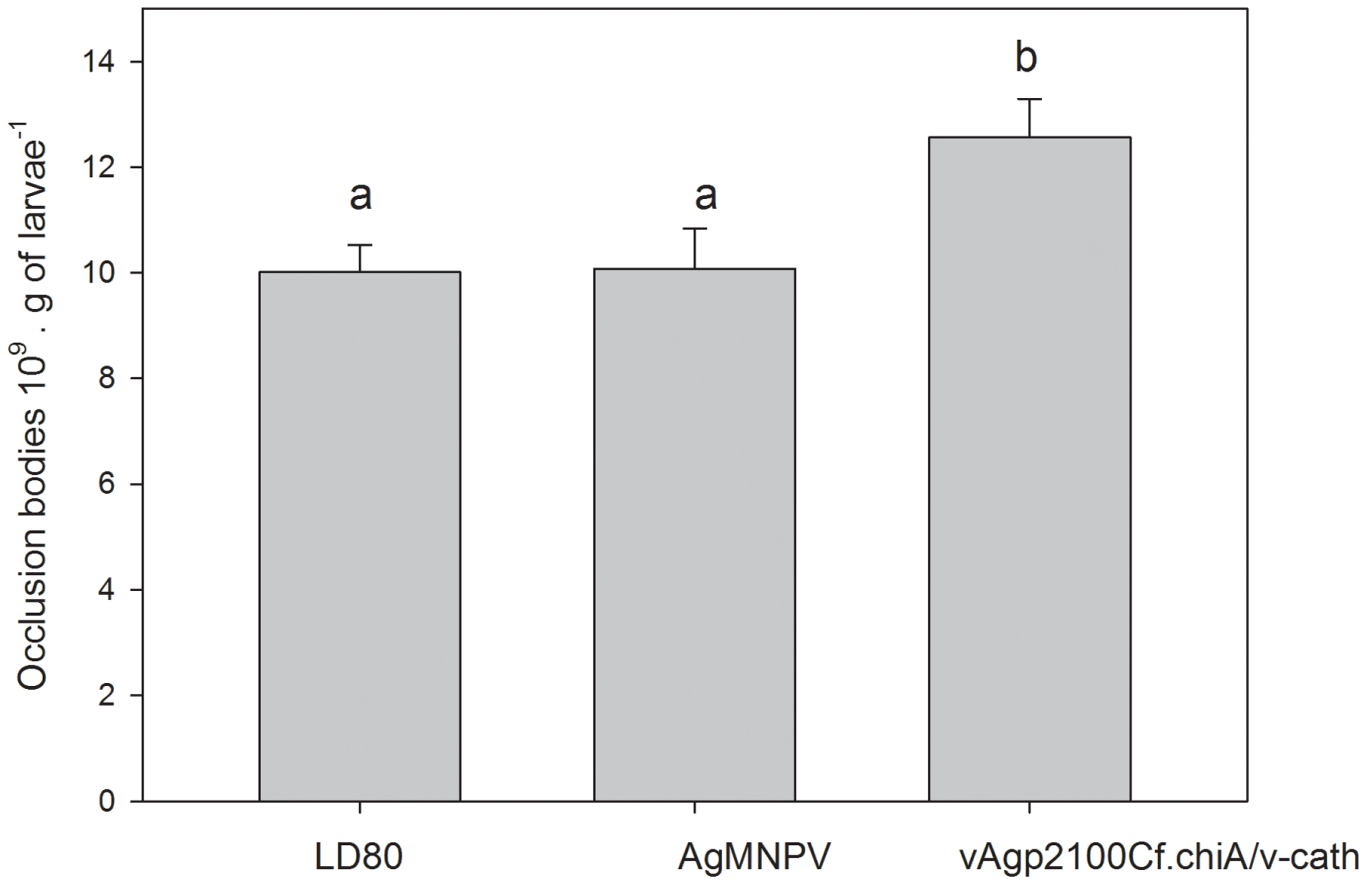
Amount of occlusion bodies (OBs) produced per gram of dead larvae infected with AgMNPV and vAgp2100Cf.chiA/v-cath. *A. gemmatalis* larvae (3^rd^ instar) were infected with AgMNPV and vAgp2100Cf.chiA/v-cath with different doses of virus and upon death, the larvae were collected and the amount of OBs per gram of dead larvae were counted in a hemacytometer. Comparisons of the numbers of occlusion bodies ×10^9^ per g of larvae ± SEM were done with parametric analysis ANOVA and means compared by Tukey test using SigmaPlot version 11.0, from Systat Software, Inc., San Jose California USA, www.sigmaplot.com.

The bioassay with 3^rd^ instars larvae showed a significant difference in LC_50_ values for the recombinant and wild type viruses. The LC_50_ values were 2.7 to 2.8 times lower for the recombinant virus when compared to the wild-type viruses AgMNPV-2D and AgMNPV (LDB80), respectively (Table 1). We also analyzed the RP for vAgp2100Cf.chiA/v-cath (RP = 2.80, CL = 1.97–3.97) with respect to the wild type isolates AgMNPV LDB80 (RP = 1) and AgMNPV 2D (RP = 1.04, 95% CL = 0.67–1.61). The recombinant virus also showed a statistically significant increase in virulence when compared to the wild type viruses. The mean time to death (MTD) values for 3^rd^ instar *A. gemmatalis* larvae infected with 4860 OBs/mL at 10 days post infection, showed a decrease of 8.4% in MTD for the recombinant virus (7.30 days) when compared to the wild type viruses (7.97). Furthermore, the amount of occlusion bodies produced in recombinant virus-infected *A. gemmatalis* larvae was significantly higher (mean ± SEM  = 12,559×10^9^ OBs/g of dead larvae ± 0.73) than wild type viruses-infected insects (AgMNPV LDB80 mean ± SEM  = 10,009×10^9^OBs of dead larvae ±0.52 and AgMNPV, mean ± SEM  = 10,077×10^9^OBs of dead larvae ±0.76) (Figure 4).

**Table 1:** LC_50_ values, relative potencies and mean time to death for wild type and recombinant virus using *A. gemmatalis* 3^rd^ instar larvae.

Virus	No. of insect	LC50 (OB/ml)	Slope ± (SE)	χ^2^	DF	RP	95% CL	MTD 4860 OBs/mL
**vAgp2100Cf.chiA/v-cath**	873	723.65	1.314±0.085	5.72	4	2.80	1.97–3.97	7.30
		(521.96–1034.51)						
**AgMNPV**	865	1942.49	0.947±0.076	4.82	4	1.04	0.67–1.61	7.97
		(1249.10–3500.30)						
**AgMNPV (LDB80)**	876	2023.22	1.087±0.083	2.99	4	1	–	7.97
		(1556.56–2756.87)						

**LC_50_**: Letal Concentration in 50% of the larvae, in occlusion bodies/mL ^−1^ **CL**: confidence limits at 95% **SE**: standard error ***X*** **^2^**: qui-square **DF**: degree of freedom **RP**: relative potencies **MTD**: Mean time to death in days.

### Increased catepsin and chitinase activities in recombinant virus-infected insect cells and insects

**Figure 5:**
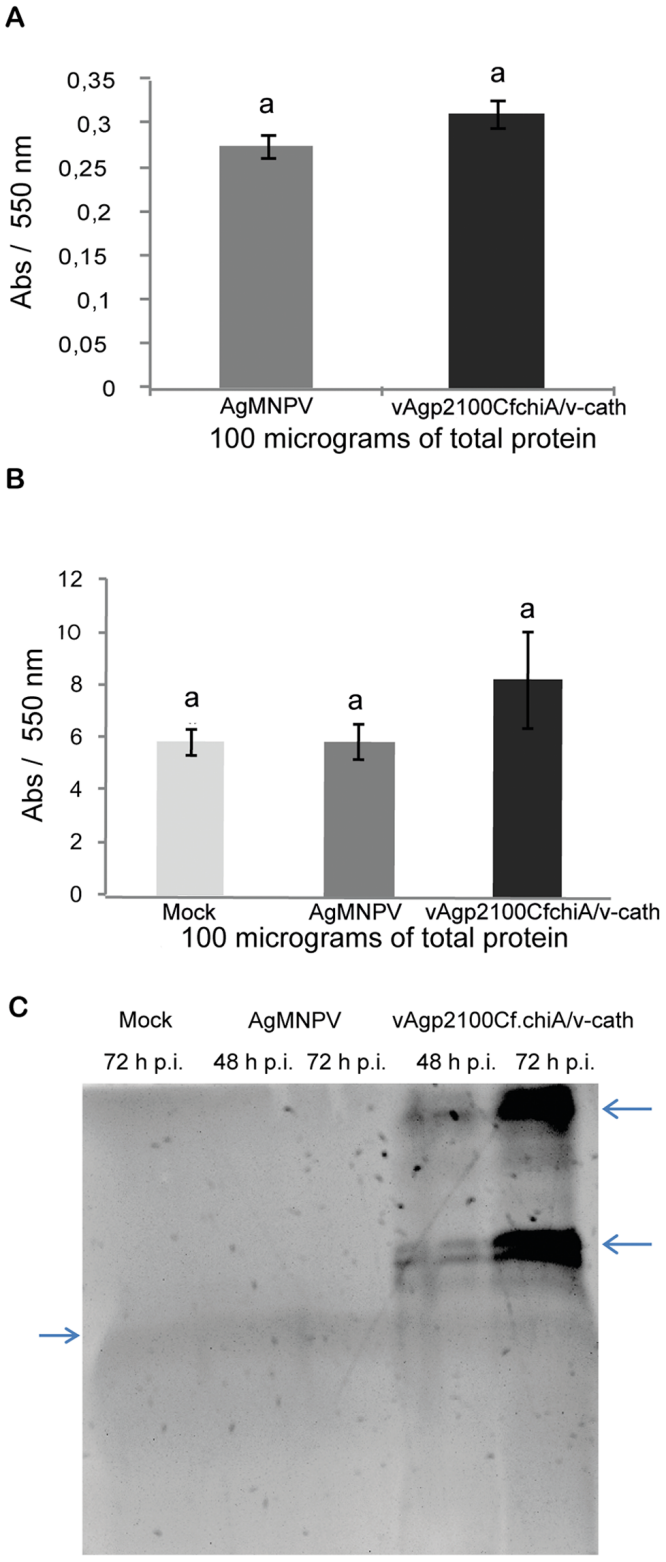
Chitinase activity analysis. (A) Chitinase activity detected in 100 micrograms of total protein from purified polyhedra of AgMNPV and vAgp2100Cf.chiA/v-cath using the DNS method [34]. (B) Chitinase activity in wild type and recombinant-virus infected insect cell extracts (100 micrograms of total protein) measured using the [4MU-(GlcNAc) 2] substrate. (C) After electrophoresis in a native polyacrylamide gel the chitinase activity was measured using chitin glycol (1%) substrate. In both assays, the recombinant-virus infected insect cell extracts showed higher chitinase activity when compared with mock infected and wild type infected insect cells extracts. It is possible to see a faint band in all lanes of the gel shown in C (large arrow), which could explain the endogenous chitinase activity detected in mock and wild type-infected insect cells extracts. The arrow heads shows the chitinase activity detected only in recombinant virus infected insect cells extracts. All assays carried out in triplicate. Tukey Test (*P*<0.05). Abs – Absorbance (550 nm).

The chitinase activities of AgMNPV and vAgp2100Cf.chiA/v-cath polyhedra were not significantly different (Figure 5A). In vAgp2100Cf.chiA/v-cath-infected UFL-AG-286 extracts (72 h p.i.) higher levels (A_595_ = 8.23±1.823) of chitinase activity were detected when compared with AgMNPV-infected UFL-AG-286 extracts (A_595_ = 5.86±0.689) and mock infected cells (A_595_ = 5.83±0.526) (Figures 5B and C) (*P*>0.05). Detection of chitinase activity in uninfected cells can be attributed to endogenous enzyme activity detected in the host cell (Figure 5B). The chitinase activity gel under native condition showed the presence of chitinase activity in vAgp2100Cf.chiA/v-cath-infected UFL-AG-286 cell extracts at 48 and 72 h p.i. (Figure 5C). A light band was detected in all samples (see arrow in Figure 5C) indicating an endogenous chitinase activity in UFL-AG-286 cells. AgMNPV-infected UFL-AG-286 cell extracts did not show the same dark bands present in vAgp2100Cf.chiA/v-cath-infected UFL-AG-286 cell extracts (see arrowheads in Figure 5C).

**Figure 6:**
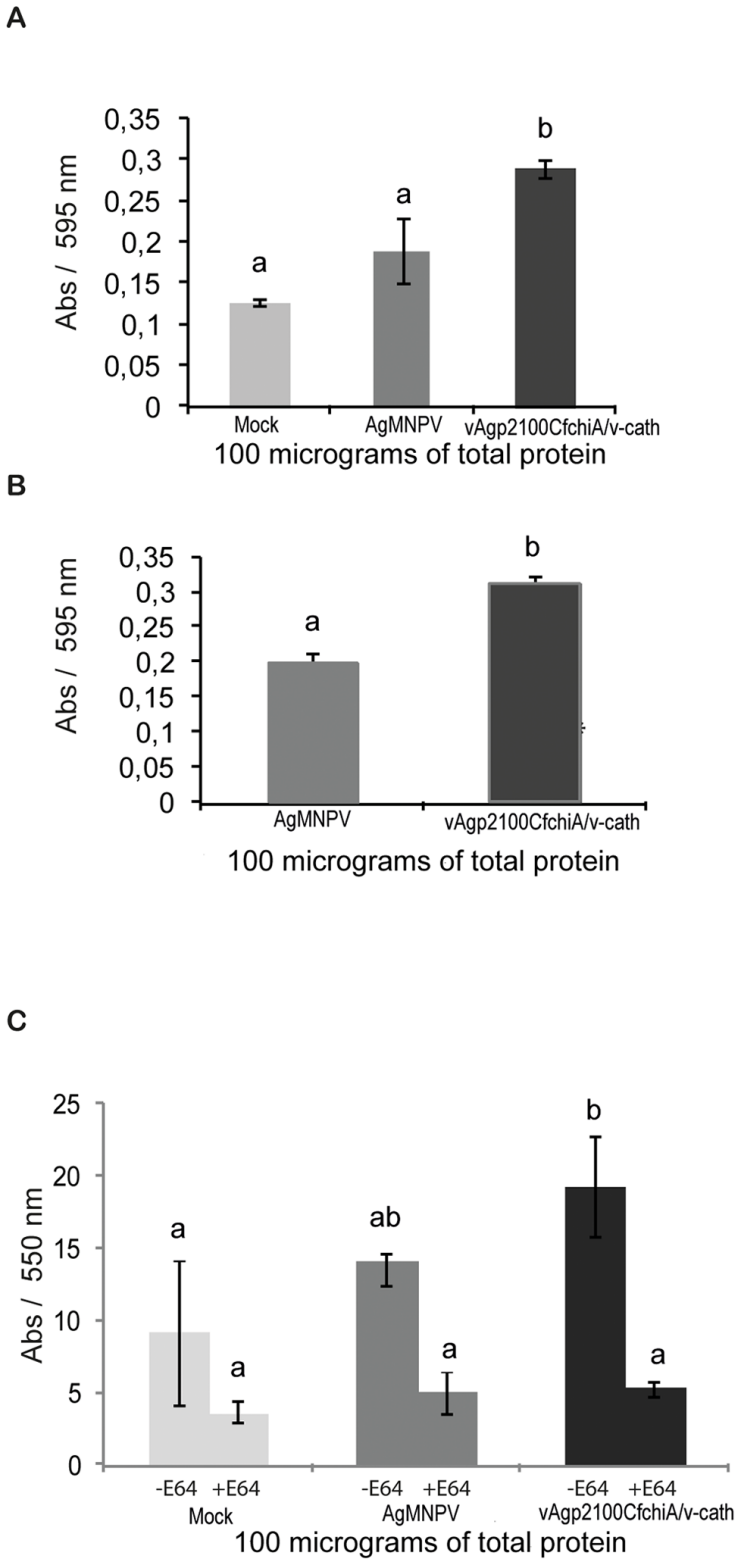
Cysteine protease activity analysis. (A) Proteolytic activity detected in 100 micrograms of total protein from hemolymph of uninfected- (mock), infected AgMNPV- and vAgp2100Cf.chiA/v-cath–insect larvae using keratin blue as substrate. The hemolymph of recombinant virus infected larvae showed an increased proteolytic activity when compared to wild type and uninfected insects (B) Proteolytic activity detected detected in 100 micrograms of total protein from purified polyhedra of AgMNPV and vAgp2100Cf.chiA/v-cath using keratin blue as substrate. Polyhedra from vAgp2100Cf.chiA/v-cath showed increased porteolytic activity when compared to AgMNPV polyhedra (C) The cysteine protease activity of uninfected UFL-AG-286 cells extract (mock), AgMNPV and vAgp2100Cf.chiA/v-cath-infected UFL-AG-286 cells extract (40 h p.i.) were measured in the presence or absence of a cysteine protease inhibitor (E-64). All extracts showed a reduction in the protease activity level, indicating the presence of cysteine proteases in all samples. However, the level of activity in the absence of E-64 was significantly higher in vAgp2100Cf.chiA/v-cath-infected UFL-AG-286 cell extracts when compared to uninfected UFL-AG-286 cells and AgMNPV-infected insect cell extracts. All assays were carried out in triplicate. Tukey Test (*P*<0.05). Abs – Absorbance.

The proteolytic activities in the hemolymph of uninfected and virus-infected *A. gemmatalis* larvae as well as in polyhedra from wild type and recombinant virus viruses were measured. The proteolytic activity detected in the hemolymph of vAgp2100Cf.chiA/v-cath-infected larvae was significantly higher (A_595_ = 0.29±0.011) than the activity detected in the hemolymph from uninfected (A_595_ = 0.12±0.004) and AgMNPV-infected (A_595_ = 0.18±0.039) larvae (*P*<0.05) (Figure 6A). Furthermore, vAgp2100Cf.chiA/v-cath polyhedra also had a higher proteolytic activity (A_595_ = 0.31±0.007) when compared to AgMNPV polyhedra (A_595_ = 0.19±0.011) (Figure 6B) (*P*<0.05). Cells infected with AgMNPV and vAgp2100Cf.chiA/v-cath viruses were further analyzed (40 h p.i.) for the presence of specific cysteine protease activity. A higher proteolytic activity in cells infected with the recombinant virus (A_280_ = 19.25±1.43) was observed when compared to cells infected with the wild-type virus (A_280_ = 13.51±1.15) and uninfected cells (A_280_ = 9.12±1.05), during the absence of the inhibitor E-64 (Figure 6C). All samples showed a decrease in the levels of proteolytic activity when analyzed in the presence of cysteine protease inhibitor E-64 (Figure 6C) (*P*<0.05).

## Discussion

Late in a baculovirus infection, infected insect larvae showed a sagging of the body and an extremely fragile cuticle. Degradation of protein components from the basal membrane occurs, and the insect body disintegrate releasing virus in the environment [7]. In the present study we have tested the hypothesis that the introduction of chitinase (*chiA*) and cathepsin (*v-cath*) genes from CfDefNPV baculovirus into the AgMNPV genome is able to induce the liquefaction and melanization of *A. gemmatalis* larvae soon after death. These genes are present in different baculovirus genomes, at a genomic region highly conserved among the species belonging to Group I of *Alphabaculovirus* [37], [38]. The *v-cath* gene was first identified in the AcMNPV genome during the nucleotide sequence analysis from the region upstream of *gp67* (also named *gp64*) gene [37], [39]. The coded protein of this gene was shown to have homology to a cysteine protease of the papain family (cathepsin), and was named V-CATH [10]. Lauzon et al., 2005 [37] also identified a *v-cath* gene in the genome of the baculovirus CfDefNPV, which codes for a protein with 76.2% identity to the amino acid sequence of V-CATH of AcMNPV, and 88.6% amino acid identity to the V-CATH of *Choristoneura fumiferana multiple nucleopolyhedrovirus* (CfMNPV). Hawtin et al., 1995 [39] were the first to identify an ORF in a baculovirus (AcMNPV) genome which coded for a protein with homology to chitinases of different organisms, particularly to a chitinase from *Serratia marcescens*, a soil gram-negative bacteria (60.5% identity), suggesting the hypothesis of horizontal transfer of a bacterial gene to the virus genome. The *chiA* from CfDefNPV was shown to have 79.3% identity to its homolog in AcMNPV [40].

Before the conclusion of the AgMNPV genome sequencing, previous studies have indicated the absence of *chiA* and *v-cath* genes near the locus of the *gp64* gene in the genome of this baculovirus [41]. However, only after the complete sequencing of the AgMNPV genome, it was possible to confirm the absence of these two genes [11]. Some studies have shown that the activity of V-CATH depends on the expression of CHIA, in such way that in the absence of the AcMNPV *chiA* gene, the proV-CATH (V-CATH active form precursor) from AcMNPV is not processed to its mature form, forming insoluble aggregates within infected cells [12], [16]. *chiA* genes from baculoviruses are considered late viral genes that encode proteins belonging to the glycosyl hydrolases family 18 [42]. Saville et al., 2004 [42] have shown that deletion of the ER retention motif (KDEL) present in *chiA* C-terminus was enough to alter its location in the cell, from cytoplasmic to the extracellular fraction during infection. Slack et al., 1995 [10] showed the temporal expression of AcMNPV V-CATH protein and confirmed its intracellular location and late expression. Hodgson et al., 2011 [43] demonstrated that the preppro-V-CATH presents a signal peptide region responsible for entry into the ER, suggesting an interaction between CHIA and proV-CATH proteins inside this organelle and thus assisting in cellular retention of proV-CATH. The analysis of *chiA* and *v-cath* transcripts in insect cells infected with AcMNPV and vAgp2100Cf.chiA/v-cath by qRT-PCR showed the presence of both transcripts from early to late phases of infection (Figure 2 A and B). Hodgson et al., 2007 [44] have shown by Northern blot that mRNAs of AcMNPV *chiA* and *v-cath* genes were expressed from 9 to 48 h p.i. in Sf21 insect cells and that the rate of intracellular CHIA accumulation during AcMNPV infection followed the same pattern observed for transcription of the *chiA* gene but with a delay of about 6 h (from 15 to 48 h p.i.). They also detected *chiA*-specific transcripts by RT-PCR, as early as 0 h p.i. (1 h after the virus was incubated with the cells).

The insertion of heterologous proteases and chitinases genes in the AcMNPV genome has been shown to improve its insecticidal activity [46],[47],[48]. Thus, Hodgson et al., 2007 [44] suggested that by the simply changing expression profile from *chiA* gene during viral infection, it would be possible to increase its virulence. In this work, the recombinant virus constructed, vAgp2100Cf.chiA/v-cath, containing the *v-cath* and *chiA* genes under the control of their original promoters, was able to decrease the LC_50_ and MTD for 3^rd^ instar *A. gemmatalis* larvae (Tables 1) when compared to the wild type virus. The expression of these genes in the infected larvae may increase the degradation of the peritrophic membrane in the midgut of the host insect allowing more viruses across it. This could result in a lower concentration of the recombinant virus required to kill the insect host. *A. gemmatalis* larvae infected with vAgp2100Cf.chiA/v-cath showed soon upon death, signs of cuticle degradation, which is characterized by a viscous liquid mass, and also melanization of larval cuticle, which is noticed by a black color. In contrast, larvae infected with the wild virus AgMNPV showed no degradation and melanization of the body cuticle soon after death. This result suggests that absence of *v-cath* and *chiA* genes in the genome of AgMNPV is associated with the loss of ability to liquefy the virus-infected larvae. On the other hand, Hawtin et al., 1997 [12], have shown that the deletion of *chiA* and *v-cath* genes from AcMNPV genome had no significant effect on LD_50_ against second instar *Trichoplusia ni* (Hübner, [1803]) larvae.

Daimon et al., 2006 [17] demonstrated that the activity of cysteine proteases was low in extracts from infected BmN insect cells, and in the hemolymph of *Bombyx mori* L. larvae infected with a recombinant BmNPV containing the *chiA* gene deleted. They also demonstrated that the activity of cysteine proteases was recovered by the BmNPV *chiA* gene when directed by the polyhedrin promoter. The absence of specific cysteine protease activity (V-CATH) in *T. ni* cells infected with AgMNPV has been demonstrated by Slack et al., 2004 [40] and contrasted with significant amounts of proteolytic activity observed in AcMNPV-infected cells.

In the present study, we have shown an increased proteolytic activity in cells and insects infected with the recombinant virus when compared to cells and insects infected with the wild type virus. Furthermore, recombinant virus purified polyhedra also showed increased proteolytic activity when compared to wild type polyhedra. This increased proteolytic activity is probably due to the expression of V-CATH during virus infection (Figure 6). The proteolytic activity in insect cells infected with AgMNPV was not significantly different from mock infected cells. Therefore, this activity is probably due to the presence of other cellular proteases [49].

The chitinase activity detected in purified polyhedra of vAgp2100Cf.chiA/v-cath was not significantly different from AgMNPV polyhedra. However, a higher chitinase activity was detected in recombinant virus-infected UFL-AG-286 cells when compared to wild-type virus-infected and uninfected cells (Figure 5B). The detection of chitinase activity in purified AgMNPV polyhedra can be explained by the presence of chitinases produced by the host larvae and incorporated in polyhedra during the occlusion phase of infection [49]. Chitinase activity in recombinant-virus infected cells was also shown by PAGE under non-denaturing conditions. The activity was more intense at 72 h p.i. which is consistent with previous studies that have shown the expression of CHIA late in infection [12], [50].

The amount of polyhedra produced in recombinant virus-infeted *A. gemmatalis* larvae were shown to be higher that wild type-infected larvae (Figure 4). Therefore, it is possible that the presence of *v-cath* and *chiA* genes in the genome of baculoviruses besides being involved in the spread of polyhedra in nature, it also enhance the amount of virus produced upon insect death. Wang et al., 2005 [50] constructed a mutant BmNPV with the *chiA* gene deleted (Bm*chi*A). The deletion of this gene resulted in the delay of cell lysis and a decrease in the amount of polyhedra produced by the Bm*chi*A- mutant when compared to cells infected with the wild virus. Insect larvae infected with Bm*chi*A- showed less degradation of the body upon death, when compared to larvae infected with the wild type virus. Daimon et al., 2006 [17] also have shown a lack of body liquefaction in *B. mori* larvae infected with a BmNPV with the *chi*A gene deleted. Another study has been reported that the *fp25k* product of BmNPV is required for post-mortem host degradation, but the mechanism by which it regulates host degradation is still unknown. The disruption of BmNPV *fp25K* attenuates the expression of *v-cath* gene at a late stage of infection, reducing the secretion of its product V-CATH [51].

In this work, *A. gemmatalis* larvae infected by recombinant viruses containing *v-cath* and *chiA* genes from CfDEFNPV showed melanization and cuticle degradation in late stages of infection (168 h p.i.), which was not observed in AgMNPV-infected larvae (Figure 3). These results confirmed that the expression of genes *v-cath* and *chiA* during viral infection can promote body degradation and melanization of *A. gemmatalis* larvae. Furthermore, the expression of these genes during the viral infection was able to enhance AgMNPV virulence. One of the main problems with the use of virus to control insect pests is their low speed of kill. Different strategies have been used to increase baculovirus speed of kill by introducing insect toxin or protease genes into the virus genome [52], [53], [54], [44], [55], [46] and/or changing baculovirus infection cycle by deleting key virus genes or introducing other genes that may alter the physiology and/or the defense mechanisms of the host insect [52], [53], [56], [57], [58]. AgMNPV have been successfully used in Brazil to control the soybean caterpillar for more than three decades. It's use had a peak around 10 years ago (2002/2003 season) with more that 2 million hectares of soybean treated with AgMNPV [59]. We have previously shown [56] that an AgMNPV with the Ecdysteroid UDP-Glycosyl Transferase (EGT) gene inactivated reduced the infected-insect feeding and time to death. Therefore, the combination of the *egt* inactivation with the introduction of the *chiA* and *v-cath* genes from CfDefNPV might further improve the insecticidal properties of this virus and become an alternative to the use of the wild type virus for the control of *A. gemmatalis*. However, we would have to take into account that the wild type virus is produced from dead larvae collected in the field. If the insects liquefy upon death, they become difficult to collect and the production of the virus would be negatively affected. In order to overcome this, the recombinant virus production would have to be carried out using insects reared in a laboratory where the dead larvae would be collected more easily than in the field.

## Supporting Information

Figure S1 **Confirmation of** ***chiA*** **and** ***v-cath*** **genes insertion into the genome of AgMNPV.** Agarose gel (0.7%) showing viral DNA (AgMNPV and vAgp2100Cf.chiA/v-cath) and plasmid DNA (p2100Cf.chiA/v-cath) *Hin*dIII restriction profiles. Lane 1– AgMNPV DNA digested with *Hin*dIII; lane 2– vAgp2100Cf.chiA/v-cath DNA digested with *Hin*dIII. lane 3– p2100Cf.chiA/v-cath plasmid DNA digested with *Hin*dIII. Lane 4– DNA fragment (3.238 bp) amplified by PCR containing the *v-cath* and *chi*A genes; lane 5– DNA probe (1.885 bp) obtained by PCR with oligonucleotides specific for the *chiA* gene. M −1 Kb plus DNA ladder marker (Invitrogen). B. Membrane containing the DNA shown in A that was hybridized with the *chiA* probe. The red arrow indicates the recombinant virus DNA fragment that hybridized with the probe (lane 2).
(TIF)

Table S1 **Nucelotide sequence of oligonucleotides used for** ***chi-A*** **and v-cath genes analysis.**
(DOCX)
