# Supplementary figures and images for: A Recombinant Anticarsia gemmatalis MNPV Harboring chiA and v-cath Genes from Choristoneura fumiferana Defective NPV Induce Host Liquefaction and Increased Insecticidal Activity
Source: PLoS One. 2013 Sep 25;8(9):e74592. doi: 10.1371/journal.pone.0074592 (PMC3783443; doi:10.1371/journal.pone.0074592)

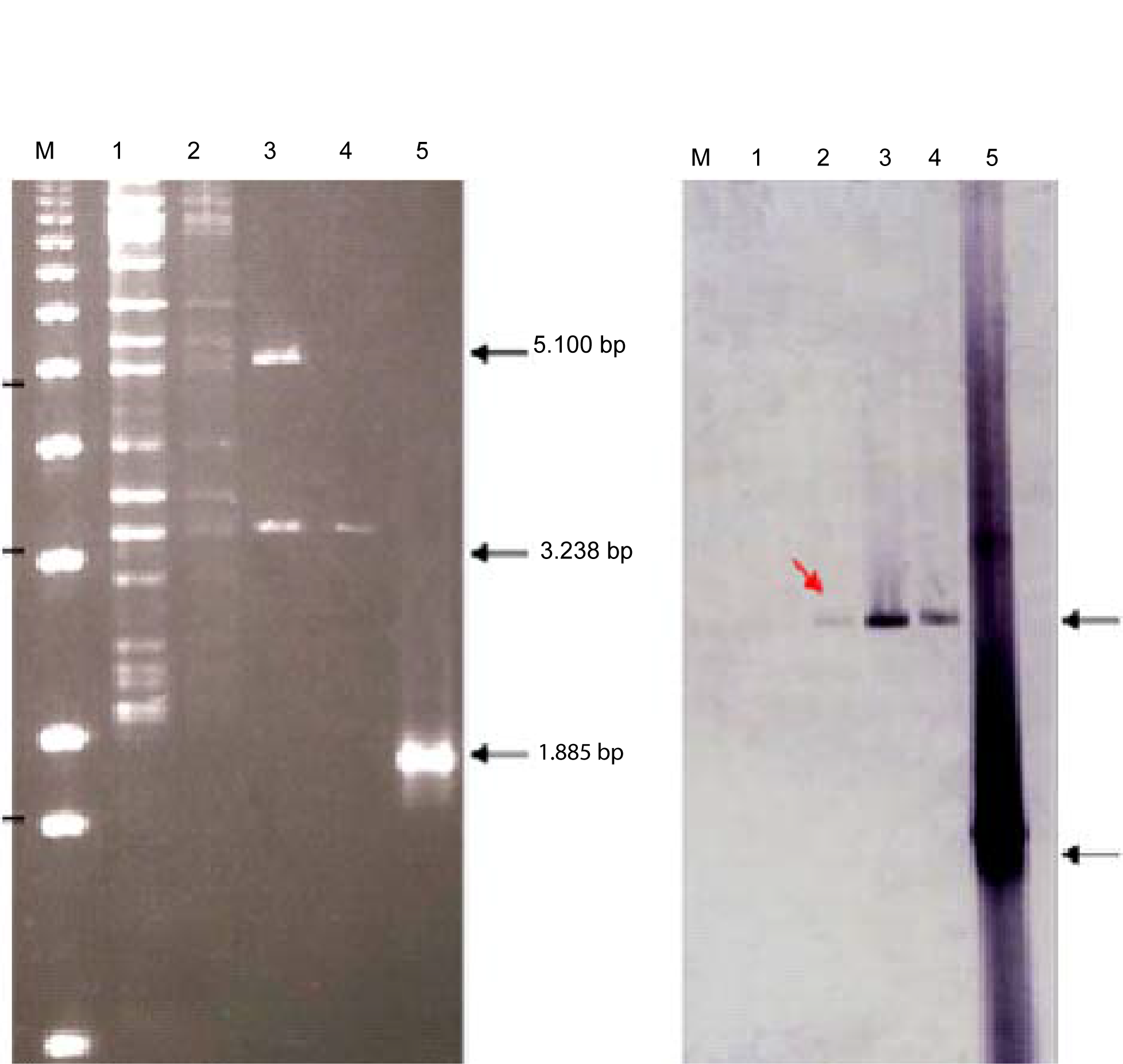

Supplement: Figure S1 — Confirmation of chiA and v-cath genes insertion into the genome of AgMNPV. Agarose gel (0.7%) showing viral DNA (AgMNPV and vAgp2100Cf.chiA/v-cath) and plasmid DNA (p2100Cf.chiA/v-cath) HindIII restriction profiles. Lane 1– AgMNPV DNA digested with HindIII; lane 2– vAgp2100Cf.chiA/v-cath DNA digested with HindIII. lane 3– p2100Cf.chiA/v-cath plasmid DNA digested with HindIII. Lane 4– DNA fragment (3.238 bp) amplified by PCR containing the v-cath and chiA genes; lane 5– DNA probe (1.885 bp) obtained by PCR with oligonucleotides specific for the chiA gene. M −1 Kb plus DNA ladder marker (Invitrogen). B. Membrane containing the DNA shown in A that was hybridized with the chiA probe. The red arrow indicates the recombinant virus DNA fragment that hybridized with the probe (lane 2). (TIF) [file pone.0074592.s001.tif]
