# Supplementary material for: A Recombinant Anticarsia gemmatalis MNPV Harboring chiA and v-cath Genes from Choristoneura fumiferana Defective NPV Induce Host Liquefaction and Increased Insecticidal Activity
Source: PLoS One. 2013 Sep 25;8(9):e74592. doi: 10.1371/journal.pone.0074592 (PMC3783443; doi:10.1371/journal.pone.0074592)
Supplement: Table S1 — Nucelotide sequence of oligonucleotides used for chi-A and v-cath genes analysis. (DOCX) [file pone.0074592.s002.docx]

**Table Supporting Information**

**Table S1**. **Nucleotide** **sequences of oligonucleotides used for *chi-A* and *v-cath* genes analysis and construction of transfer vector.**

| **Oligonucleotides** | **Nucleotide sequence (5’→3’)** |
| --- | --- |
| AcQuitF | CTGTTCCGTGAGCGAT |
| AcQuitR | CGTACACACCCCACTC |
| AcCatF | ACTGGCGTCGTCTCAAC |
| AcCatR | GATCATTTGCTGCTCCG |
| CfQuitF | GTCAAGGTGTGCGATG |
| CfQuitR | AACAAATGTGAGAGGT |
| CfCatF | CTTCCTGTCGCCATAGA |
| CfCatR | AACCCTGTTCTCCCCAG |
| QUITCf F | AGATCTCATGGTGCACTATTTGAAAGTAG |
| QUITCf R | GGATCCATGAAAACCTGACGCAGCAACTCTAAAAC |
| CATHCf F | AGATCTATGTTGTTGTTATACAAAAACGTTGTTTGG |
| CATHCf R | GGATCCTACAAATGCAGAGTGATC |
| attBQuitF | GGGGACAAGTTTGTACAAAAAAGCAGGCTTCATGGTGCACTATGGTTTTAAAG |
| attBQuitR | GGGGACCACTTTGTACAAGAAAGCTGGGTCTTAAAGGTCTTCTTTTTTAAATATG |
| attBCathF | GGGGACAAGTTTGTACAAAAAAGCAGGCTTCATGAACAAGTGTGTAATTTGTTGTAT |
| attBCathR | GGGGACCACTTTGTACAAGAAAGCTGGGTCTTAATAGATGCAGCTTTCTGAAGG |
| polAgF | GATCTGCAATAAAGATATCTAGACCGC |
| polAgR | GCGGTCTAGATATCTTTATTGCAGCTC |
| SphIF | GCATGCCGCTGTGCATGG |
| PstIR | CTGCAGGGATTTTGGGTGTG |
